# Investigation of a Novel *NTRK1* Variation Causing Congenital Insensitivity to Pain With Anhidrosis

**DOI:** 10.3389/fgene.2021.763467

**Published:** 2021-12-06

**Authors:** Kai Yang, Yi-Cheng Xu, Hua-Ying Hu, Ya-Zhou Li, Qian Li, Ying-Yi Luan, Yan Liu, Yong-Qing Sun, Zhan-Ke Feng, You-Sheng Yan, Cheng-Hong Yin

**Affiliations:** ^1^ Prenatal Diagnosis Center, Beijing Obstetrics and Gynecology Hospital, Capital Medical University, Beijing, China; ^2^ Department of Neurology, Aerospace Center Hospital, Beijing, China; ^3^ Jiaen Genetics Laboratory, Beijing Jiaen Hospital, Beijing, China; ^4^ Department of Pediatric Orthopaedic, The Third Hospital of Hebei Medical University, Shijiazhuang, China; ^5^ National Research Institute for Family Planning, Beijing, China

**Keywords:** NTRK1 gene, congenital insensitivity to pain with anhidrosis, whole-exome sequencing, metabolomic study, molecular dynamic analysis

## Abstract

**Background:** Congenital insensitivity to pain with anhidrosis (CIPA), a rare autosomal recessive sensory neuropathy, was caused mainly by biallelic mutations in the *NTRK1* gene. The pathogenesis of CIPA still needs further elucidation.

**Methods:** Here, we recruited a CIPA case and introduced whole-exome sequencing (WES) to identify the causative variation. Subsequently, an *in silico* molecular dynamic (MD) analysis was performed to explore the intramolecular impact of the novel missense variant. Meanwhile, *in vitro* functional study on the novel variant from a metabolomic perspective was conducted *via* the liquid chromatography–mass spectrometry (LC-MS) approach, of which the result was verified by quantitative real-time PCR (qRT-PCR).

**Results:** A novel compound heterozygous variation in *NTRK1* gene was detected, consisting of the c.851–33T > A and c.2242C > T (p.Arg748Trp) variants. MD result suggested that p.Arg748Trp could affect the intramolecular structure stability. The results of the LC-MS and metabolic pathway clustering indicated that the NTRK1^Arg748Trp^ variant would significantly affect the purine metabolism *in vitro*. Further analysis showed that it induced the elevation of *NT5C2* mRNA level.

**Conclusion:** The findings in this study extended the variation spectrum of *NTRK1*, provided evidence for counseling to the affected family, and offered potential clues and biomarkers to the pathogenesis of CIPA.

## 1 Introduction

Congenital insensitivity to pain with anhidrosis (CIPA, MIM #256800), also known as hereditary sensory and autonomic neuropathy IV (HSAN IV), is a rare autosomal recessive mendelian disorder mainly characterized by absence of nociception, anhidrosis, and increased *S. aureus* infection risk (Indo et al., 2008). Patients with CIPA do not feel pain from any noxious stimuli, including inflammation and heat (Goldberg et al., 2007), so they often develop finger-tip and oral cavity wounds due to self-mutilation in childhood and bony deformities due to recurrent fractures (Schon et al., 2018).

The prevalence of CIPA is currently unknown, whereas biallelic mutations in the *NTRK1* gene account for the vast majority of all cases (3). Still, there were a few CIPA cases found to be caused by mutations in *NGF* and *PRDM12* genes (Einarsdottir et al., 2004; Chen et al., 2015), suggesting the importance of differential diagnosis at molecular level. All these genes are involved in the differentiation and maturation of nociceptor, the specialized neuron for pain sensing (Drissi et al., 2020), suggesting their synergistic interaction in this process.

The *NTRK1* (MIM **#**191315) gene, encoding the neurotrophic tyrosine kinase receptor type 1 (TrkA), belongs to a family of nerve growth factor receptors whose ligands include neurotrophins (Indo, 2012), which plays a pivotal role in regulating development of both the central and peripheral neurons (Bibel and Barde, 2000). *NTRK1* is located in chromosome 1q23.1 region, spans over 25-kb (kilobase) region, and contains 17 exons (https://www.omim.org/). To date, more than 130 pathogenic variants for CIPA in *NTRK1* have been indexed (HGMD professional v2019.4, The Human Gene Mutation Database, http://www.hgmd.cf.ac.uk/ac/index.php). Among different ethnic groups, there are different hotspot variants with high incidence (Shatzky et al., 2000; Indo, 2001; Zhao et al., 2020); yet, the effects of specific *NTRK1* variants, especially missense variants, on molecular structure and protein function still deserve further investigation.

In this study, a family with a boy affected by CIPA was recruited and underwent a comprehensive clinical evaluation and genetic detection. Further investigation was conducted in two aspects. On one hand, molecular dynamic (MD) analysis was performed to predict the intramolecular impact of the novel variant and its potential biological effects; on the other hand, expression vector carrying the mutant NTRK1 cDNA was transfected into tool cell lines to discover its impact on cellular metabolic profile.

## 2 Materials and Methods

### 2.1 Subjects

A family with a 15-year-old boy exhibiting “intellectual disability, multiple fractures” was recruited in our center. We performed a comprehensive clinical evaluation on the proband.

### 2.2 Genetic Analysis

For all participants, genomic DNA was extracted from 200 μl of peripheral blood using the DNA Blood Mini Kit (Qiagen GmbH) according to the protocol of manufacturer.

Whole-exome sequencing (WES) was performed as described in previous study (Yang et al., 2019). Briefly, DNA fragments were hybridized and captured by xGenExome Research Panel of IDT (Integrated DNA Technologies, San Diego, United States). Libraries were tested for enrichment by quantitative fluorescence PCR, and the size distribution and concentration were determined using Agilent Bioanalyzer 2100 (Agilent Technologies, Santa Clara, CA, United States). The NovaSeq 6000 platform (Illumina, San Diego, United States), along with 150-bp pair-end reads, was used for the sequencing of DNA with ∼300pM per sample using the NovaSeq Reagent Kit (Illumina). Sequencing raw reads (with the quality level Q30% > 90%) were aligned to the human reference genome (accession no. hg19/GRCh37, ftp://hgdownload.cse.ucsc.edu/goldenPath/hg19/chromosomes/) using the Burrows–Wheeler Aligner tool, and PCR duplicates were removed by using Picard v1.57 (http://picard.sourceforge.net/). Variant calling was performed with the Verita Trekker^®^ Variants Detection system (v 2.0, Berry Genomics, Inc., China) and Genome Analysis Toolkit (https://software.broadinstitute.org/gatk/) and then annotated and interpreted using *in silico* tools of ANNOVAR (v 2.0) and Enliven^®^ Variants Annotation Interpretation systems (Berry Genomics, Inc.) (Wang et al., 2010) on the basis of the common guideline by ACMG (American College of Medical Genetics and Genomics) (Richards et al., 2015).

Sanger sequencing was performed as validation method for suspected variations. The conservatism of amino acid (AA) affected by missense variant was analyzed using MEGA7 (http://www.megasoftware.net) with default parameters. In addition, the pathgenecity of missense variant was predicted with Revel (an ensemble method for predicting the pathogenicity of missense variants using the following tools individually: MutPred, FATHMM, VEST, PolyPhen, SIFT, PROVEAN, MutationAssessor, MutationTaster, LRT, GERP, SiPhy, phyloP, and phastCons; with the cutoff value of >0.7) as in our previous study (Hu et al., 2021).

### 2.3 Structural Modeling and Molecular Dynamic Simulation

SWISS-MODEL tool (https://swissmodel.expasy.org/) was used to model the wild-type (WT) and mutant (Mut) domains using 4F0I template covering the NTRK1:p.Arg748 site (http://www1.rcsb.org/).

We then ran the MD simulation for both models. The program CHARMM22 was used to add hydrogen atoms, N- and C-terminal patches to the models (Hu et al., 2021). The generated models were solvated and neutralized in a box with TIP3P water at a minimum of 13 Å between the model and the wall of the box. All simulations were run using NAMD 2.9 with periodic boundary conditions applied (Desiderio et al., 2019). The temperature was held at 300 K, whereas the pressure was controlled at 1 atm. The time step was set to 2 fs, the particle mesh Ewald method was applied to model the electrostatics, and the van der Waals interactions cutoff was set at 12 Å. Both models followed a three-step pre-equilibration totaling 600 ps, the last snapshots of which were chosen as the starting structures for 20-ns productive simulations without constraints.

### 2.4 Plasmids Construction, Cell Culture, and Transfection

To explore the functional impact on cellular metabolism by the novel missense variant, expression plasmid vectors were constructed using a lentiviral backbone (pLV-hef1a-mNeongreen-P2A-Puro-WPRE-CMV-MCS-3×flag) containing the WT or Mut (with variant c.2242C > T) *NTRK1* cDNA (see detailed data in Supplementary Material S1).

HEK-293 (human embryonic kidney) cell lines were purchased commercially (SyngenTech Inc., Beijing, China) and cultured by regular means with DMEM medium +10% BSA (Invitrogen, United States). The plasmid vectors were transfected using Lipofectamine 3000 (Thermo Fisher Scientific, United States). Forty-eight hours later, the intensity of green fluorescent protein (GFP) was observed by fluorescence microscope and the expression of *NTRK1* was detected by quantitative real-time PCR (qRT-PCR) with ABI 7500 system (Thermo Fisher Scientific, United States) to determine the transfection effect (detailed methods in Supplementary Material S1).

### 2.5 Metabonomic Analysis

The cells were submitted to metabonomic analysis 48 h after transfection. The ultra-performance liquid chromatography combined with quadrupole time-of-flight tandem mass spectrometry analysis was performed on Nexera X2 system (Shimadzu, Japan) coupled with a TripleTOF 5600 quadrupole-time-of-flight mass spectrometer (AB SCIEX, United States). Liquid chromatography separation was performed on a ZORBAX Eclipse Plus C18 column (2.1 × 100 mm, 3.5 μm, Agilent, United States) maintained at 45°C. Independent reference lock mass ions *via* Analyst TF 1.6 and MarkerView 1.2.1 were used to ensure mass accuracy during data acquisition. The assigned modified metabolite ions were identified by database searches in the HMDB (http://www.hmdb.ca/spectra/ms/search) databases. The mass tolerance for the HMDB database search was set at 0.05 Da.

#### 2.5.1 Statistical Analysis

Mann–Whitney U test were first performed to compare the WT (no. pHS-AVC-LY059) group with Mut (no. pHS-AVC-LY 060) groups. Orthogonal projection to latent structure–discriminant analysis (PLS-DA) was used to determine the distributions and find the metabolic differences between the two groups using MetaboAnalyst 5.0 (http://www.metaboanalyst.ca/MetaboAnalyst/). The PLS-DA models were cross-validated using a 10-fold method with unit variance scaling. The parameter R2 was used to evaluate the fitting condition for the PLS-DA models, and Q2 was used to assess predictive ability. Negative or very low Q2 values indicated that the differences between groups were not statistically significant. The PLS-DA model removes variation in the X matrix that is not correlated with the Y matrix. Thus, normally, only one predictive component is used for the discrimination between two classes.

#### 2.5.2 Pathway Analysis

Chemical metabolites were analyzed using the MetaboAnalyst web portal for pathway analysis and visualization (http://www.metaboanalyst.ca/). Pearson correlations were used to evaluate relationships between metabolites (*p* < 0.05 and impact > 0.01 were considered significant).

#### 2.5.3 qRT-PCR

It was used to detect the mRNA relative expression level of those metabolic enzymes that were suspectedly affected (detailed data shown in Supplementary Material S1).

## 3 Results

### 3.1 Clinical Manifestations

**FIGURE 1:**
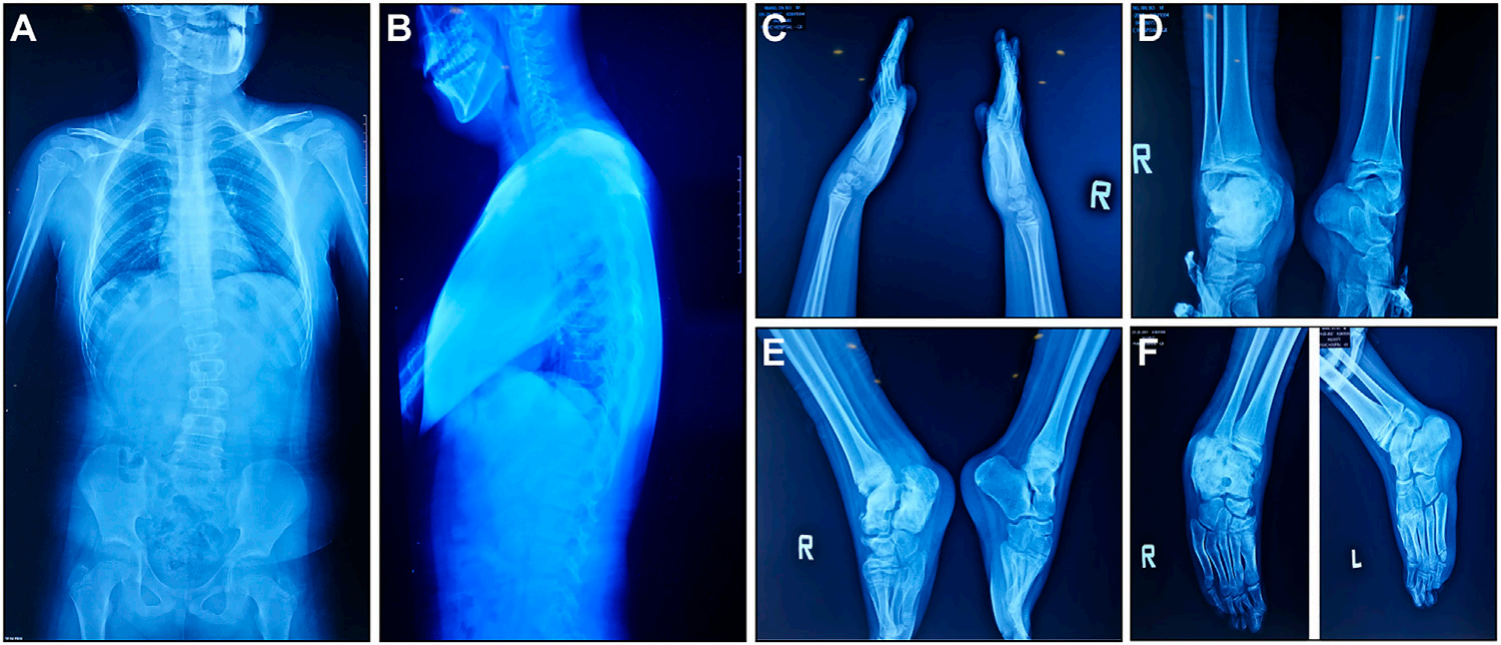
X-ray manifestations of the recruited CIPA patient. **(A,B)** Front and side images of the torso. **(C)** Hands and wrists. **(D–F)** Ankles in different angles.

According to medical history survey, the patient has developed bilateral ankle swelling and fractures since 18 months. His intellectual development was significantly behind peers in motor and language development. Multiple fractures to his right ankle and swelling of his ankle and knee limited his movement. X-ray result showed scoliosis, osteomyelitis-like changes in the right ankle, bone deformation, bone destruction on multiple joints, and osteomyelitis-like changes after partial vertebral deformation (Figure 1).

### 3.2 Variation Analysis of the NTRK1 Gene

**FIGURE 2:**
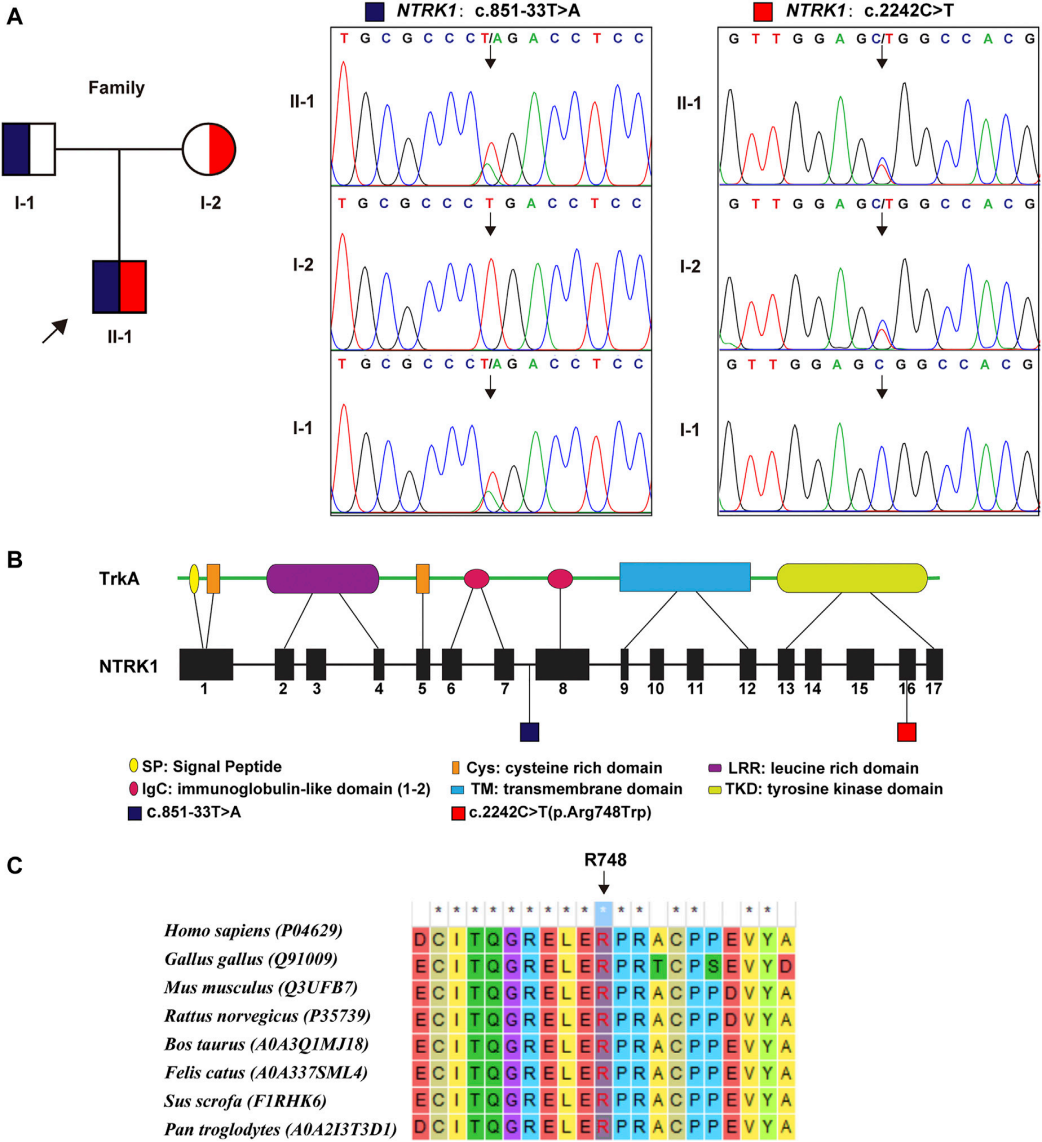
Genetic findings. **(A)** Pedigree diagram and the two variants detected in this case. Dark blue and red blocks represent the carrying status of these variants, respectively. **(B)** The location of these two variant illustrated in gene and protein schematics. **(C)** The conservatism of the amino acid Arg748 (R748) affected by c. 2242C > T variant across species.

WES identified a compound heterozygous variation in the *NTRK1* gene, consisting of two variant, namely, (NM_002529.3) c.851–33T > A and c.2242C > T (p. Arg748Trp) (Figure 2A). Figure 2B showed the location of the two variants in the *NTRK1* gene and peptide chain schematics. Among these two, the former is prevalent in East-Asian populations, whereas the latter is an unreported novel missense variant. The revel score was 0.650 indicating that this variant was uncertain but likely to be pathogenic (Supplementary Table S1 in Supplementary Material S1).

The Arg748 residue maintained evolutionary conserved among species (Figure 2C). To elucidate the effect of p. Arg748Trp on molecular structure and protein function, we conducted MD simulation and *in vitro* experiments. The results are as follows.

### 3.3 Intramolecular Impact of the NTRK1: p.Arg748Trp Variant

**FIGURE 3:**
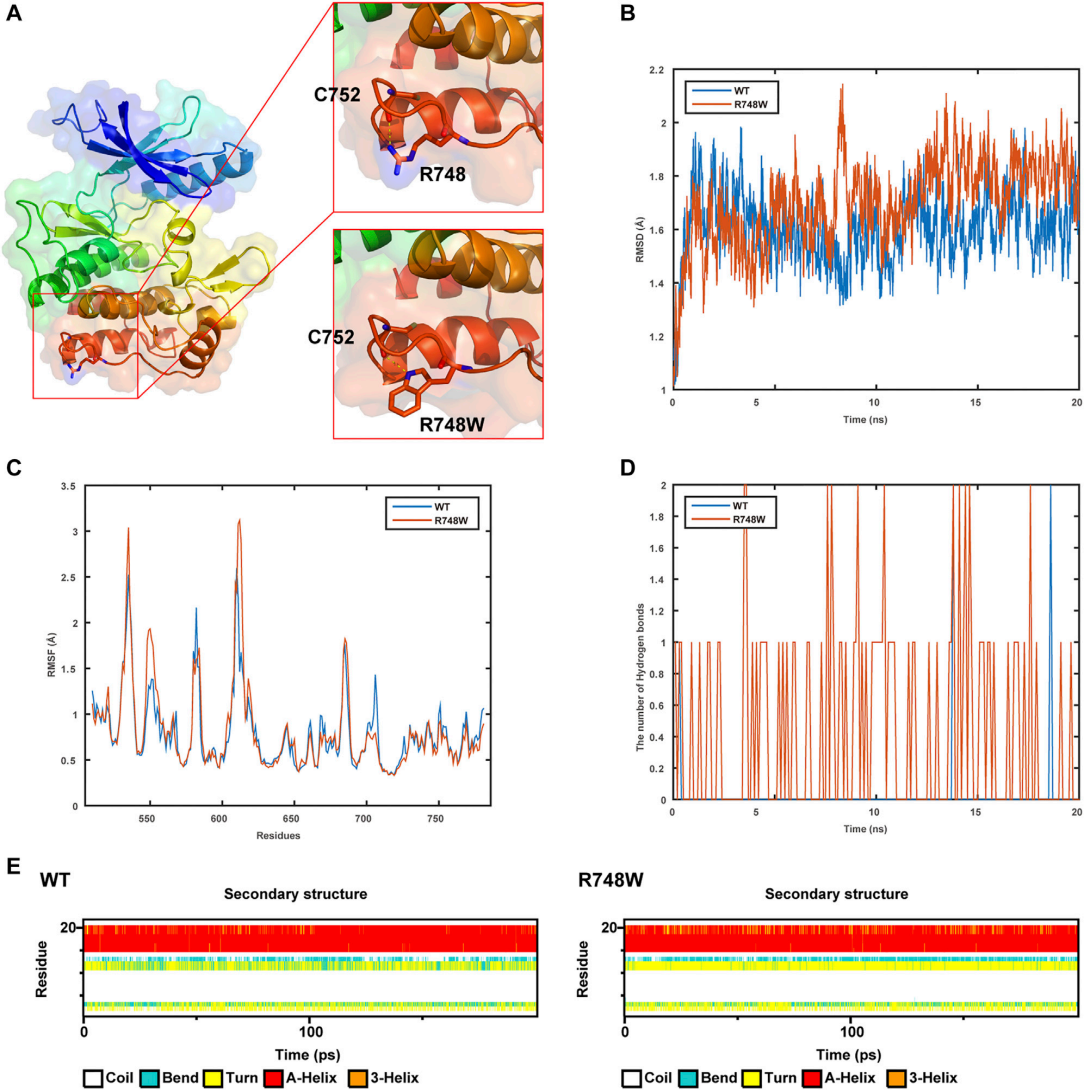
Results of structure modeling and molecular dynamic simulation. **(A)** The structures of domain containing Arg748 (R748) or the Arg748Trp (R748W) mutant. Hydrogen bonds formed between the Arg748/Arg748Trp and Cys752 residues are shown in stick representation. The dotted yellow lines represent the hydrogen bonds involving Arg748. **(B)** The trajectory of RMSD (C*α*) (root mean square deviation) of the two proteins. **(C)** RMSF (root mean square fluctuation) of the two proteins calculated from each simulation. **(D)** The number of hydrogen bonds formed between Arg748 (WT)/Arg748Trp (R748W)and the rest residues in the two protein models, respectively. **(E)** Secondary structural components of corresponding region in the two models (WT and R748W mutant) as a function of time.

The WT and Mut models were shown in Figure 3A. It was indicated that the p.Arg748Trp variant replacing the strongly basic arginine by a large AA with benzene rings broke the hydrogen bonds formed by the side chain of Arg748 and expectantly changed its potential distribution.

As shown by MD, it indicated that the Arg748Trp Mut was more flexible than the WT according to the trajectory of root mean square deviation and root mean square fluctuation (Figures 3B,C). Besides, the hydrogen bonds formed between Trp748, and the rest residues were more than those between Arg748 and the rest (Figure 3D), which might cause the corresponding loop to be less flexible. Moreover, Arg748Trp could influence the secondary structure of the connection region between two β sheets (Figure 3E).

### 3.4 Metabolic Impact of the NTRK1^Arg748Trp^ Mutant

**FIGURE 4:**
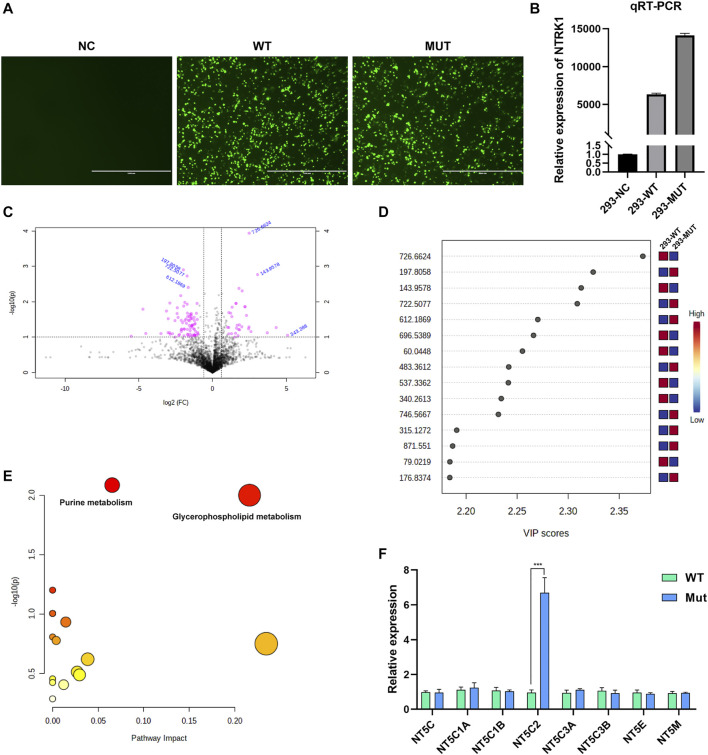
Results of *in vitro* study. **(A)** The GFP fluorescent signal at 48 h after transfection. NC, no vector; WT, with wild-type MFN2 cDNA plasmid; MUT, with MFN2: c.638T > C mutant cDNA plasmid. Scale bar, 1000 μm; magnification, ×100. **(B)** The relative *NTRK1*mRNA levels in three cell groups. **(C)** The volcano plot showing significantly different compounds. Red dots represent the compounds with difference >2.0 folds and *p* < 0.05, blue font represents the m/z molecular weight information. **(D)** Part of the compounds with vip (variable importance in projection) value > 1. The X- and Y-axes stand for vip score and m/z, respectively. **(E)** KEGG pathway enrichment result. Each point represents a pathway, with X- and Y-axes indicating the importance of a compound related to the pathway and-log10(P) value, respectively. **(F)** The relative mRNA levels of various enzymes that can catalyze the hydrolysis of the AMP in WT and Mutant transfected cell groups.

According to the intracellular fluorescence signal of GFP (Figure 4A) and the relative expression level of *NTRK1* mRNA (Figure 4B), the transfection efficiency was ideal.

Metabolites with significant difference between the two groups (WT, Lab No. pHS-AVC-LY061; Mut, pHS-AVC-LY062) after transfection were demonstrated in Figures 4C,D, and also listed in Supplementary Table S1 (the screening criteria for compounds with difference were FC > 2.0 or < 0.5, *p* value <0.05, vip >1. FC, fold count; vip, variable importance in projection). Pathway clustering result indicated that the metabolic pathway most significantly affected by NTRK1^Arg748Trp^ Mut was the purine metabolism (Figure 4E; Table 1). To be specific, the volume of AMP declined, whereas that of adenosine increased (Supplementary Table S1). Subsequently, among all enzymes catalyzing the hydrolysis of the AMP, qRT-PCR revealed a significant up-regulation of *NT5C2* (5-Ppime-Nucleotidase, Cytosolic II, MIM #600417) mRNA expression in the Mut-transfected cells compared to WT cells (Figure 4F).

**TABLE 1:** Metabolic pathways that significantly affected by the NTRK1^Arg748Trp^ variant (in reverse order of comprehensive significance).

KEGG (kyoto encyclopedia of genes and genomes)	Total	Hits	Raw p	Impact
Purine metabolism	65	4	0.0082243	0.06541
Glycerophospholipid metabolism	36	3	0.010002	0.21631
Linoleic acid metabolism	5	1	0.062952	0
Taurine and hypotaurine metabolism	8	1	0.098896	0
Sulfur metabolism	8	1	0.098896	0
Primary bile acid biosynthesis	46	2	0.11678	0.01446
Alpha-linolenic acid metabolism	13	1	0.15591	0
Glycosylphosphatidylinositol (GPI)–anchor biosynthesis	14	1	0.16689	0.00399
Nicotinate and nicotinamide metabolism	15	1	0.17774	0.23465
Sphingolipid metabolism	21	1	0.24006	0.03854
Glutathione metabolism	28	1	0.3071	0.02698
Porphyrin and chlorophyll metabolism	30	1	0.3252	0.02955
Glycine, serine and threonine metabolism	33	1	0.3515	0
Arachidonic acid metabolism	36	1	0.37683	0
Arginine and proline metabolism	38	1	0.39319	0.01212
Drug metabolism-cytochrome P450	55	1	0.5167	0

*Total, the total number of compounds in the pathway; Hits, the actually matched number from the user uploaded data; Raw p, the original *p* value calculated from the enrichment analysis; Impact, the pathway impact value calculated from pathway topology analysis.

## 4 Discussion

Pain is essential in teaching us how to use our bodies optimally and avoid or respond to injuries while being permanently painless results in a significant morbidity and mortality (Drissi et al., 2020). The normal development and function of nociceptors is essential for pain sensation, and at least, nine genes have been recognized to play crucial roles in these progresses (Drissi et al., 2020). Among them, NGF, TrkA (*NTRK1*), and Prdm12 (*PRDM12*) collaboratively participate in the promotion of nociceptor fate (Desiderio et al., 2019); correspondingly, pathogenic variations in these genes result in autosomal recessive conditions with similar symptoms, namely, HSAN V (MIM #608654), HSAN IV (CIPA), and HSAN VIII (MIM #616488).

In this study, the patient displayed typical CIPA manifestations such as multiple fractures, ankle swelling, intellectual delay, rachioscoliosis, and osteomyelitis. The subsequent WES detection identified the causative compound heterozygous *NTRK1*variation consisting of c.851–33T > A and c.2242C > T (p. Arg748Trp) (NM_001012331). As we stated previously, the former variant is a hotspot mutation in East Asian people (Indo, 2001; Wang et al., 2017; Liu et al., 2018; Li et al., 2019; Zhao et al., 2020; Li et al., 2021); whereas the latter one is a novel missense variant. According to the common interpretation guidelines provided by ACMG (14), it was recognized as “uncertain significance” (with evidences of PM1+PM2+PP2+BP4). The BP4 evidence was due to the “uncertain” result by Revel and benign computational verdict by several tools in the Varsome predicting website (https://varsome.com/). Therefore, further investigation was necessary to confirm the pathogenicity of this variant.

The conservatism of Arg748 AA residue across species indirectly suggested the pathogenicity of p. Arg748Trp variant. Besides, according to the structural modeling and MD simulation result, the p.Arg748Trp variant might change the conformation of the loop which the Arg748 belongs to (Bertrand et al., 2012). Thus, the variant could probably cause the structural instability of TrkA protein and further broke its binding to NGF. Yet, more mechanistic experiments are needed to validate whether this variant could cause loss of protein function.

With regard to the *in vitro* study, we intended to analyze the cellular effect of NTRK1^Arg748Trp^ variant from the perspective of metabolic changes. It was demonstrated that the purine metabolism pathway was significantly affected. Furthermore, it seemed that the up-regulation of *NT5C2* mRNA level should be a major contribution to this effect. Duarte et al*.* demonstrated that NT5C2 could participate in the neural development and motility of *Drosophila* by regulating the AMPK signaling pathway (Duarte et al., 2019). Singgih et al*.* showed that impairment in cNT5-II (NT5C2 orthologs in *Drosophila*) resulted in disease-relevant behaviors in *Drosophila*. Novarino et al*.* revealed that *NT5C2* mutations could lead to a severe neurodegenerative motor neuron disease, hereditary spastic paraplegia (Spastic paraplegia 45, MIM #613162) (Novarino et al., 2014), which was validated by other studies (Darvish et al., 2017; Naseer et al., 2020). Collectively, it can be deduced that NT5C2 plays a crucial role in the development and functional maintenance of neurosystem. Up to our knowledge, the NGF/TrkA signaling could participate in the process of cell survival, proliferation, and migration through at least three pathways, namely, RAS/RAF/MEK/ERK, PI3K/Akt, and PLC/PCK (Thomaz et al., 2020; Esteban-Villarrubia et al., 2020). Through *in silico* analysis, we found that there were multiple binding sites of transcriptional factors such as STAT1β, ELK1, and NF-κB at the upstream promoter region of *NT5C2* gene (Supplementary Material 1), and all these factors are associated with at least one of the above pathways (27). That is to say, NGF/TrkA could probably regulate *NT5C2* transcription *via* one of these signaling pathways and jointly involved in the development of neurons; yet, detailed functional experiments are also needed to verify this hypothesis.

The future pregnancy of this couple will have a 25% chance of getting affected, so potential interventions such as prenatal diagnosis or pre-implantation diagnosis need to be recommended. The major limitation of our study was that the evidences were still not solid to elucidate the functional impact of the novel *NTRK1* variant. As a necessary supplement, the alteration of interaction between NGF and TrkA by it and how TrkA regulate NT5C2 should be clarified in our further investigation.

In summary, we present with a novel compound heterozygous *NTRK1* variation including one novel variant, c.2242C > T (p. Arg748Trp). The findings in this study extend the *NTRK1* mutation spectrum and provide critical information for genetic counseling to guide the future pregnancy of the family. In addition, a potential mechanism of how TrkA relates NT5C2 was also suggested, which might be essential in the development of neurons.

## Data Availability

The sequence data presented in the study are deposited in the repository at: https://doi.org/10.6084/m9.figshare.16934713.v1.
