# Supplementary material for: Investigation of a Novel NTRK1 Variation Causing Congenital Insensitivity to Pain With Anhidrosis
Source: Front Genet. 2021 Dec 6;12:763467. doi: 10.3389/fgene.2021.763467 (PMC8686761; doi:10.3389/fgene.2021.763467)
Supplement: Supplementary file 1 [file DataSheet3.PDF]

Supplementary material 1

1. Supplementary material for section 3.4 "Metabolic impact of the NTRK1^{Arg748Trp} mutant"

1.1 Detailed data of the vector construction and cell transfection.

(1) cDNA sequences.

WT: pHS-AVC-LY061; pLV-hef1a-mNeongreen-P2A-Puro-WPRE-CMV-NTRK1(Human, NM_002529)-3Xflag

5'-ATGCTGCGAGGCGGACGGCGCGGGCAGCTTGGCTGGCACAGCTGGGCTGCGGGGCCGGGCAGCCTGCTGGCTTGGCTGATACTGGCATCTGCGGG
CGCCGCACCCTGCCCCGATGCCTGCTGCCCCCACGGCTCCTCGGGACTGCGATGCACCCGGGATGGGGCCCTGGATAGCCTCCACCACCTGCCCGGCG
CAGAGAACCTGACTGAGCTCTACATCGAGAACCAGCAGCATCTGCAGCATCTGGAGCTCCGTGATCTGAGGGGCCTGGGGGAGCTGAGAAACCTCAC
CATCGTGAAGAGTGGTCTCCGTTTCGTGGCGCCAGATGCCTTCCATTTCACTCCTCGGCTCAGTCGCCTGAATCTCTCCTTCAACGCTCTGGAGTCTCT
CTCCTGGAAAACTGTGCAGGGCCTCTCCTTACAGGAACTGGTCCTGTCGGGGAACCCTCTGCACTGTTCTTGTGCCCTGCGCTGGCTACAGCGCTGGG
AGGAGGAGGGACTGGGCGGAGTGCCTGAACAGAAGCTGCAGTGTCATGGGCAAGGGCCCCTGGCCCACATGCCCAATGCCAGCTGTGGTGTGCCCA
CGCTGAAGGTCCAGGTGCCCAATGCCTCGGTGGATGTGGGGGACGACGTGCTGCTGCGGTGCCAGGTGGAGGGGCGGGGCCTGGAGCAGGCCGGCT
GGATCCTCACAGAGCTGGAGCAGTCAGCCACGGTGATGAAATCTGGGGGTCTGCCATCCCTGGGGCTGACCCTGGCCAATGTCACCAGTGACCTCAA
CAGGAAGAACGTGACGTGCTGGGCAGAGAACGATGTGGGCCGGGCAGAGGTCTCTGTTCAGGTCAACGTCTCCTTCCCGGCCAGTGTGCAGCTGCA
CACGGCGGTGGAGATGCACCACTGGTGCATCCCCTTCTCTGTGGATGGGCAGCCGGCACCGTCTCTGCGCTGGCTCTTCAATGGCTCCGTGCTCAATG
AGACCAGCTTCATCTTCACTGAGTTCCTGGAGCCGGCAGCCAATGAGACCGTGCGGCACGGGTGTCTGCGCCTCAACCAGCCCACCCACGTCAACAA
CGGCAACTACACGCTGCTGGCTGCCAACCCCTTCGGCCAGGCCTCCGCCTCCATCATGGCTGCCTTCATGGACAACCCTTTCGAGTTCAACCCCGAGG
ACCCCATCCCTGTCTCCTTCTCGCCGGTGGACACTAACAGCACATCTGGAGACCCGGTGGAGAAGAAGGACGAAACACCTTTTGGGGTCTCGGTGGC
TGTGGGCCTGGCCGTCTTTGCCTGCCTCTTCCTTTCTACGCTGCTCCTTGTGCTCAACAAATGTGGACGGAGAAACAAGTTTGGGATCAACCGCCCGG
CTGTGCTGGCTCCAGAGGATGGGCTGGCCATGTCCCTGCATTTCATGACATTGGGTGGCAGCTCCCTGTCCCCCACCGAGGGCAAAGGCTCTGGGCTC
CAAGGCCACATCATCGAGAACCCACAATACTTCAGTGATGCCTGTGTTCACCACATCAAGCGCCGGGACATCGTGCTCAAGTGGGAGCTGGGGGAGG
GCGCCTTTGGGAAGGTCTTCCTTGCTGAGTGCCACAACCTCCTGCCTGAGCAGGACAAGATGCTGGTGGCTGTCAAGGCACTGAAGGAGGCGTCCGA
GAGTGCTCGGCAGGACTTCCAGCGTGAGGCTGAGCTGCTCACCATGCTGCAGCACCAGCACATCGTGCGCTTCTTCGGCGTCTGCACCGAGGGCCGC
CCCCTGCTCATGGTCTTTGAGTATATGCGGCACGGGGACCTCAACCGCTTCCTCCGATCCCATGGACCTGATGCCAAGCTGCTGGCTGGTGGGGAGGAT

GTGGCTCCAGGCCCCCTGGGTCTGGGGCAGCTGCTGGCCGTGGCTAGCCAGGTCGCTGCGGGGATGGTGTACCTGGCGGGTCTGCATTTTGTGCACCG
GGACCTGGCCACACGCAACTGTCTAGTGGGCCAGGGACTGGTGGTCAAGATTGGTGATTTTGGCATGAGCAGGGATATCTACAGCACCGACTATTACC
GTGTGGGAGGCCGCACCATGCTGCCCATTCGCTGGATGCCGCCCGAGAGCATCCTGTACCGTAAGTTCACCACCGAGAGCGACGTGTGGAGCTTCGGC
GTGGTGCTCTGGGAGATCTTCACCTACGGCAAGCAGCCCTGGTACCAGCTCTCCAACACGGAGGCAATCGACTGCATCACGCAGGGACGTGAGTTGG
AGCGGCCACGTGCCTGCCCACCAGAGGTCTACGCCATCATGCGGGGCTGCTGGCAGCGGGAGCCCCAGCAACGCCACAGCATCAAGGATGTGCACGC
CCGGCTGCAAGCCCTGGCCCAGGCACCTCCTGTCTACCTGGATGTCCTGGGC-3'

Mut: pHS-AVC-LY062; pLV-hef1a-mNeongreen-P2A-Puro-WPRE-CMV-NTRK1(Human, NM_002529, 2242C>T)-3Xflag
5'-ATGCTGCGAGGCGGACGGCGCGGGCAGCTTGGCTGGCACAGCTGGGCTGCGGGGCCGGGCAGCCTGCTGGCTTGGCTGATACTGGCATCTGCGGG
CGCCGCACCCTGCCCCGATGCCTGCTGCCCCCACGGCTCCTCGGGACTGCGATGCACCCGGGATGGGGCCCTGGATAGCCTCCACCACCTGCCCGGCG
CAGAGAACCTGACTGAGCTCTACATCGAGAACCAGCAGCATCTGCAGCATCTGGAGCTCCGTGATCTGAGGGGCCTGGGGGAGCTGAGAAACCTCAC
CATCGTGAAGAGTGGTCTCCGTTTCGTGGCGCCAGATGCCTTCCATTTCACTCCTCGGCTCAGTCGCCTGAATCTCTCCTTCAACGCTCTGGAGTCTCT
CTCCTGGAAAACTGTGCAGGGCCTCTCCTTACAGGAACTGGTCCTGTCGGGGAACCCTCTGCACTGTTCTTGTGCCCTGCGCTGGCTACAGCGCTGGG
AGGAGGAGGGACTGGGCGGAGTGCCTGAACAGAAGCTGCAGTGTCATGGGCAAGGGCCCCTGGCCCACATGCCCAATGCCAGCTGTGGTGTGCCCA
CGCTGAAGGTCCAGGTGCCCAATGCCTCGGTGGATGTGGGGGACGACGTGCTGCTGCGGTGCCAGGTGGAGGGGCGGGGCCTGGAGCAGGCCGGCT
GGATCCTCACAGAGCTGGAGCAGTCAGCCACGGTGATGAAATCTGGGGGTCTGCCATCCCTGGGGCTGACCCTGGCCAATGTCACCAGTGACCTCAA
CAGGAAGAACGTGACGTGCTGGGCAGAGAACGATGTGGGCCGGGCAGAGGTCTCTGTTCAGGTCAACGTCTCCTTCCCGGCCAGTGTGCAGCTGCA
CACGGCGGTGGAGATGCACCACTGGTGCATCCCCTTCTCTGTGGATGGGCAGCCGGCACCGTCTCTGCGCTGGCTCTTCAATGGCTCCGTGCTCAATG
AGACCAGCTTCATCTTCACTGAGTTCCTGGAGCCGGCAGCCAATGAGACCGTGCGGCACGGGTGTCTGCGCCTCAACCAGCCCACCCACGTCAACAA
CGGCAACTACACGCTGCTGGCTGCCAACCCCTTCGGCCAGGCCTCCGCCTCCATCATGGCTGCCTTCATGGACAACCCTTTCGAGTTCAACCCCGAGG
ACCCCATCCCTGTCTCCTTCTCGCCGGTGGACACTAACAGCACATCTGGAGACCCGGTGGAGAAGAAGGACGAAACACCTTTTGGGGTCTCGGTGGC
TGTGGGCCTGGCCGTCTTTGCCTGCCTCTTCCTTTCTACGCTGCTCCTTGTGCTCAACAAATGTGGACGGAGAAACAAGTTTGGGATCAACCGCCCGG
CTGTGCTGGCTCCAGAGGATGGGCTGGCCATGTCCCTGCATTTCATGACATTGGGTGGCAGCTCCCTGTCCCCCACCGAGGGCAAAGGCTCTGGGCTC
CAAGGCCACATCATCGAGAACCCACAATACTTCAGTGATGCCTGTGTTCACCACATCAAGCGCCGGGACATCGTGCTCAAGTGGGAGCTGGGGGAGG
GCGCCTTTGGGAAGGTCTTCCTTGCTGAGTGCCACAACCTCCTGCCTGAGCAGGACAAGATGCTGGTGGCTGTCAAGGCACTGAAGGAGGCGTCCGA
GAGTGCTCGGCAGGACTTCCAGCGTGAGGCTGAGCTGCTCACCATGCTGCAGCACCAGCACATCGTGCGCTTCTTCGGCGTCTGCACCGAGGGCCGC

CCCCTGCTCATGGTCTTTGAGTATATGCGGCACGGGGACCTCAACCGCTTCCTCCGATCCCATGGACCTGATGCCAAGCTGCTGGCTGGTGGGGAGGAT
GTGGCTCCAGGCCCCCTGGGTCTGGGGCAGCTGCTGGCCGTGGCTAGCCAGGTCGCTGCGGGGATGGTGTACCTGGCGGGTCTGCATTTTGTGCACCG
GGACCTGGCCACACGCAACTGTCTAGTGGGCCAGGGACTGGTGGTCAAGATTGGTGATTTTGGCATGAGCAGGGATATCTACAGCACCGACTATTACC
GTGTGGGAGGCCGCACCATGCTGCCCATTCGCTGGATGCCGCCCGAGAGCATCCTGTACCGTAAGTTCACCACCGAGAGCGACGTGTGGAGCTTCGGC
GTGGTGCTCTGGGAGATCTTCACCTACGGCAAGCAGCCCTGGTACCAGCTCTCCAACACGGAGGCAATCGACTGCATCACGCAGGGACGTGAGTTGG
AGTGGCCACGTGCCTGCCCACCAGAGGTCTACGCCATCATGCGGGGCTGCTGGCAGCGGGAGCCCCAGCAACGCCACAGCATCAAGGATGTGCACGC
CCGGCTGCAAGCCCTGGCCCAGGCACCTCCTGTCTACCTGGATGTCCTGGGC-3'

(2) Vector backbone diagram

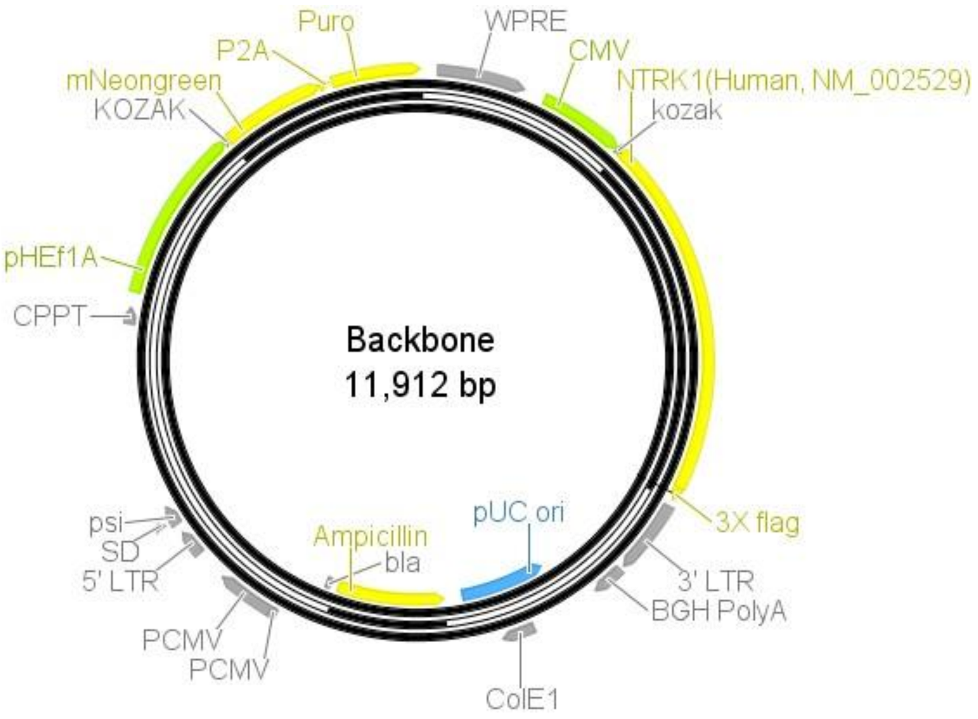

Validation by restriction endonuclease digestion:

载体酶切结果

<table>
<tr><th rowspan="2">marker</th><th colspan="3">电泳条带</th><th rowspan="2">条带注释</th></tr>
<tr><th>1</th><th>2</th><th>M</th></tr>
<tr><td>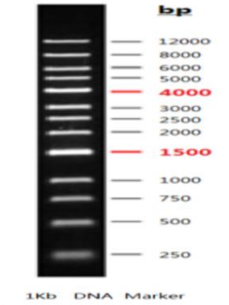</td><td colspan="3">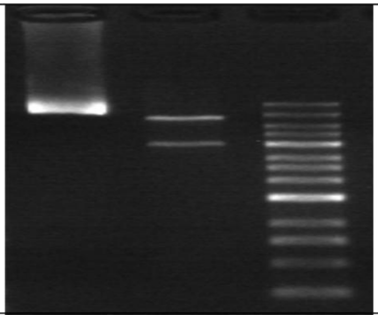</td><td>Lane 1: Plasmid pHS-AVC-LY061
Lane 2: Plasmid pHS-AVC-LY061 Digested with BsiWI-KpnI (4159bp/7761bp)
Lane M: DNA Marker</td></tr>
<tr><td>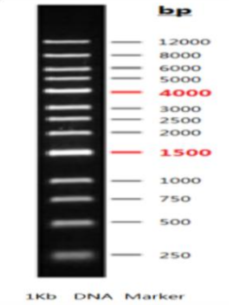</td><td colspan="3">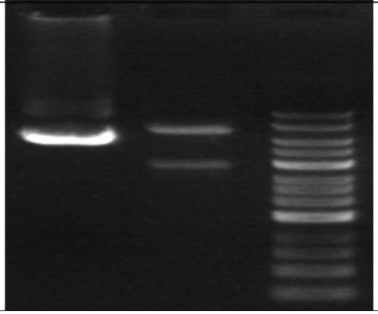</td><td>Lane 1: Plasmid pHS-AVC-LY062
Lane 2: Plasmid pHS-AVC-LY062 Digested with BsiWI-KpnI (4159bp/7761bp)
Lane M: DNA Marker</td></tr>
</table>

(3) Primers used in the plasmid vectors' construction and qRT-PCR.

PHS-AVC-LY061	H8887-SEQR: TAGCCTTGTGTGTGGTAGATCCAC
	PEGFP-N-5 :TGGGAGGTCTATATAAGCAGAG
	G0169344-1-SEQ1 :AACAGAAGCTGCAGTGTCAT

	G0169344-1-seq1r :CCCCACATCCACCGAGGCAT
	G0169344-1-SEQ2 :AACAACGGCAACTACACGCT
	G0169344-1-SEQ3: ACAAGATGCTGGTGGCTGTC
PHS-AVC-LY062	G0169344-1-seq1r :CCCCACATCCACCGAGGCAT
	G0169344-1-seq2 :TACAGCGCTGGGAGGAGGAG
	G0169344-1-seq3: CTGTCTCCTTCTCGCCGGTG
	G0169344-1-seq4: CTGGTGGGGAGGATGTGGCT
	PEGFP-N-5 :TGGGAGGTCTATATAAGCAGAG

(4) Data of qRT-PCR (48 h after transfection with NTRK1 vectors)

NTRK1	293FT	0.3	0.3	0.3	0.315	0.014	0.046
	293FT-pHS-AVC-LY061	6472.0	6338.8	6165.5	6325.445	153.701	0.024
	293FT-pHS-AVC-LY062	14362.3	14164.6	13777.2	14101.378	297.607	0.021
	293FT-pHS-BVC-LW345	1.0	1.0	1.0	1.000	0.012	0.012

$p<0.001$

(5) Reaction reagents and condition

The expression level was assessed by real-time Quantitative Fluorescence PCR using SYBR Premis Ex Taq II(Perfect Real Time) (Takara) with ABI 7500 system. Data are presented as mean±standard deviation of three independent real-time PCR experiments. The PCR cycle was as follows: 10 min 95° C, 1 cycle; 10 s 95° C, 30 s 60° C＋fluorescence acquisition, 55 cycles. Values for each gene were normalized to expression level of beta-actin gene (ACTB) via the 2-ΔΔCT method.

Primers for internal control:

ACTB-F	GGCATGGGTCAGAAGGATT
ACTB-R	TGGTGCCAGATTTTCTCCA

Melting peaks

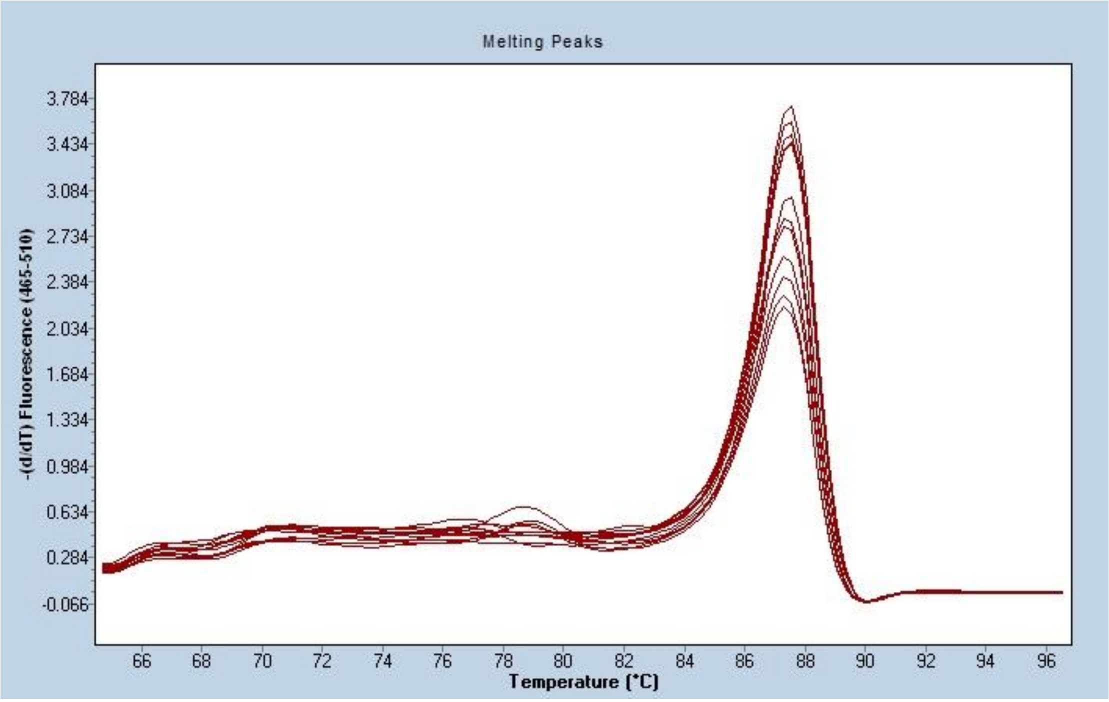

1.2 Primer sequences used to testing the mRNA expression of related metabolic enzymes

Gene	Forward	Reverse	**length**
**NT5C1B**	CTTGTGCGCAGTCAATGGTC	GTGAGGAGTCATGCAGGCTT	157
**NT5C3B**	GGGCATTGGTGATATCCTGG	TGGTTTTGCCCTCAAGTTGC	200
**NT5C2**	TGCCAGTCAAGTGATGCGTT	ATCCACTGATGTGCGGTTCC	190
**NT5C**	ACGAATTATCCTGACAAGGGACA	TCTCCCTCCAGTTGTCACTCC	191
**NT5E**	CCTGTTGGTGATGAAGTTGTGG	AAACCCGAATGTCCCAGTGC	176

**NT5C3A**	GCTGGAATCGGCGATGTACT	TCTGTATTCCTCAAGGCACCA	173
**NT5M**	GGAGCAGATTGTGCTGACCA	CAGAATGGCCTTCCAGTCGT	199
**NT5C1A**	CTTGTTTCGCATGGACGAGG	TCATGAGGACGATGTCGAAGAC	200
**NT5C1B**	GGGAGGACGAGGACGACTAC	TTTCCAGACCCTCTTGCTCG	186

The reaction condition was as in 1(5).

1.3 The potential binding sites of transcriptional factors at the up-stream promoter region of *NT5C2* gene.

0 GR [T05076]	1 TFII-I [T00824]	2 GR-beta [T01920]	3 C/EBPbeta [T00581]	4 LEF-1 [T02905]	5 C/EBPalpha [T00105]	6 NF-AT2 [T01945]	7 STAT4 [T01577]
8 c-Ets-1 [T00112]	9 RelA [T00594]	10 STAT1beta [T01573]	11 GATA-1 [T00306]	12 HNF-1A [T00368]	13 HNF-4alpha [T03828]	14 c-Myb [T00137]	15 YY1 [T00915]
16 TFIID [T00820]	17 HNF-3alpha [T02512]	18 FOXP3 [T04280]	19 POU2F2 (Oct-2.1) [T00646]	20 SRY [T00997]	21 PR B [T00696]	22 PR A [T01661]	23 TCF-4E [T02878]
24 TBP [T00794]	25 HOXD9 [T01424]	26 HOXD10 [T01425]	27 RXR-alpha [T01345]	28 RAR-beta [T00721]	29 ENKTF-1 [T00255]	30 Pax-5 [T00070]	31 p53 [T00671]
32 GR-alpha [T00337]	33 AP-2alphaA [T00035]	34 NF-1 [T00539]	35 E2F-1 [T01542]	36 Elk-1 [T00250]	37 XBP-1 [T00902]	38 NF-kappaB1 [T00593]	39 c-Jun [T00133]

40 VDR [T00885]	41 PXR-1:RXR-alpha [T05671]	42 ER-alpha [T00261]	43 ATF3 [T01313]	44 USF2 [T00878]	45 NFI/CTF [T00094]	46 IRF-2 [T01491]	47 AhR:Arnt [T05394]
48 ETF [T00270]	49 NF-AT1 [T01948]	50 E2F [T00221]	51 AP-1 [T00029]	52 c-Fos [T00123]	53 GATA-2 [T00308]	54 c-Ets-2 [T00113]	55 POU2F1 [T00641]
56 EBF [T05427]	57 IRF-1 [T00423]	58 NF-AT1 [T00550]	59 T3R-beta1 [T00851]	60 PEA3 [T00685]	61 TCF-4 [T02918]	62 MAZ [T00490]	63 GCF [T00320]
64 Sp1 [T00759]	65 AR [T00040]	66 RBP-Jkappa [T01616]	67 PPAR-alpha:RXR-alpha [T05221]	68 Ik-1 [T02702]	69 HNF-1C [T01951]	70 RAR-beta:RXR-alpha [T05420]	71 AhR [T01795]
72 NF-Y [T00150]	73 MEF-2A [T01005]						

Table S1. Results of predicting the pathogenicity of the missense variant (c.2242C>T: p.Arg748Trp) of *NTRK1*

Prediction Tool	Score	Prediction
**SIFT**	0	damaging
**Polyphen2 HDIV**	1	probably damaging
**Polyphen2 HVAR**	1	probably damaging
**LRT**	0.000050	deleterious
**MutationTaster**	1	disease_causing

**MutationAssessor**	2.23	medium
**FATHMM**	-1.73	damaging
**MetaSVM**	0.2150	damaging
**MetaLR**	0.5684	damaging
**PROVEAN**	-6.32	damaging
**M-CAP**	0.178442305215	damaging
**CADD**	7.526983	-
**CADD_Phred**	28.9	damaging
**dann**	0.999	damaging
**REVEL_score**	0.650	-
