# Supplementary figures and images for: Investigation of a Novel NTRK1 Variation Causing Congenital Insensitivity to Pain With Anhidrosis
Source: Front Genet. 2021 Dec 6;12:763467. doi: 10.3389/fgene.2021.763467 (PMC8686761; doi:10.3389/fgene.2021.763467)

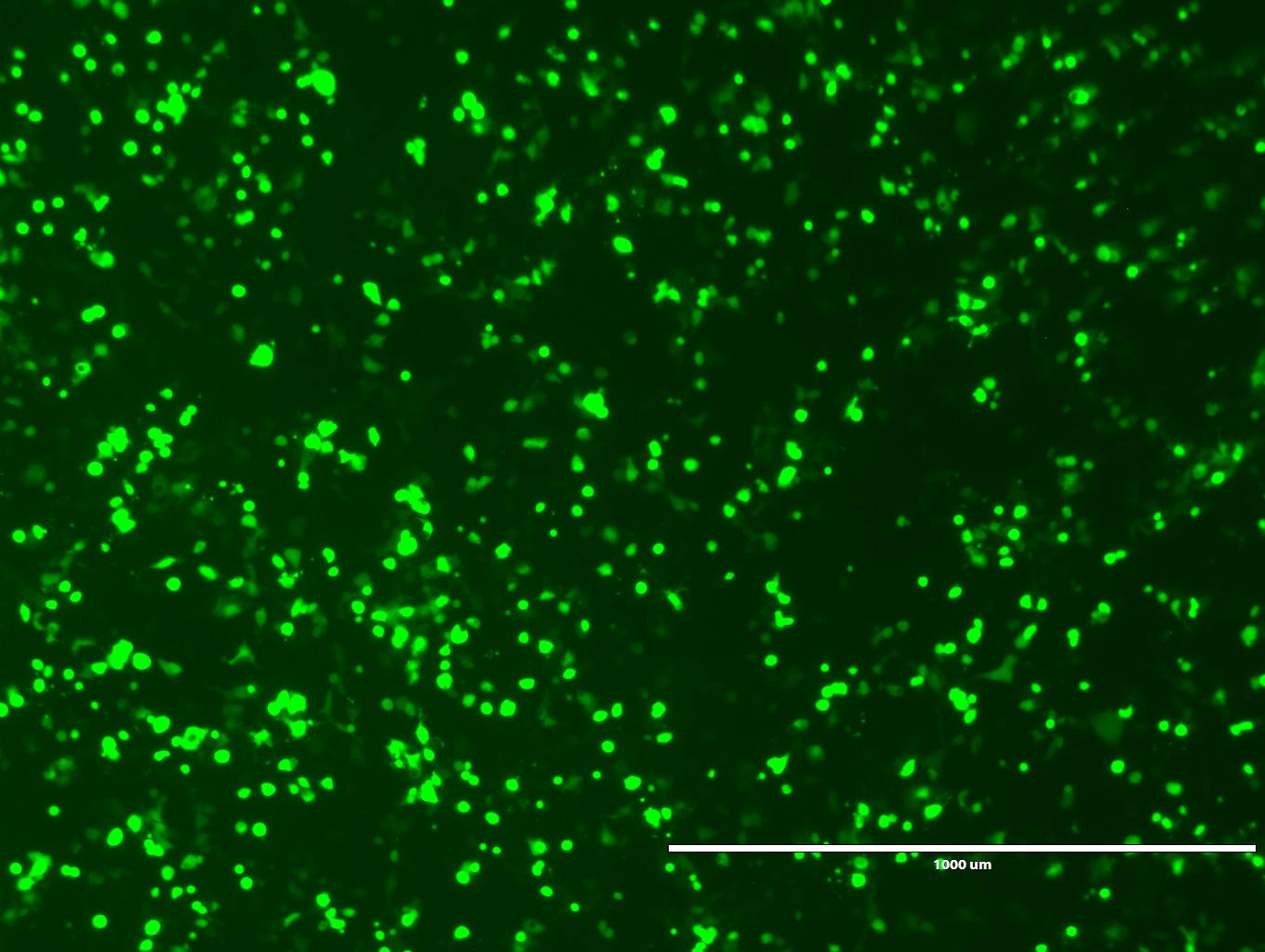

Supplement: Supplementary file 2 [file DataSheet2.ZIP › 4A raw images/MUT.jpg]

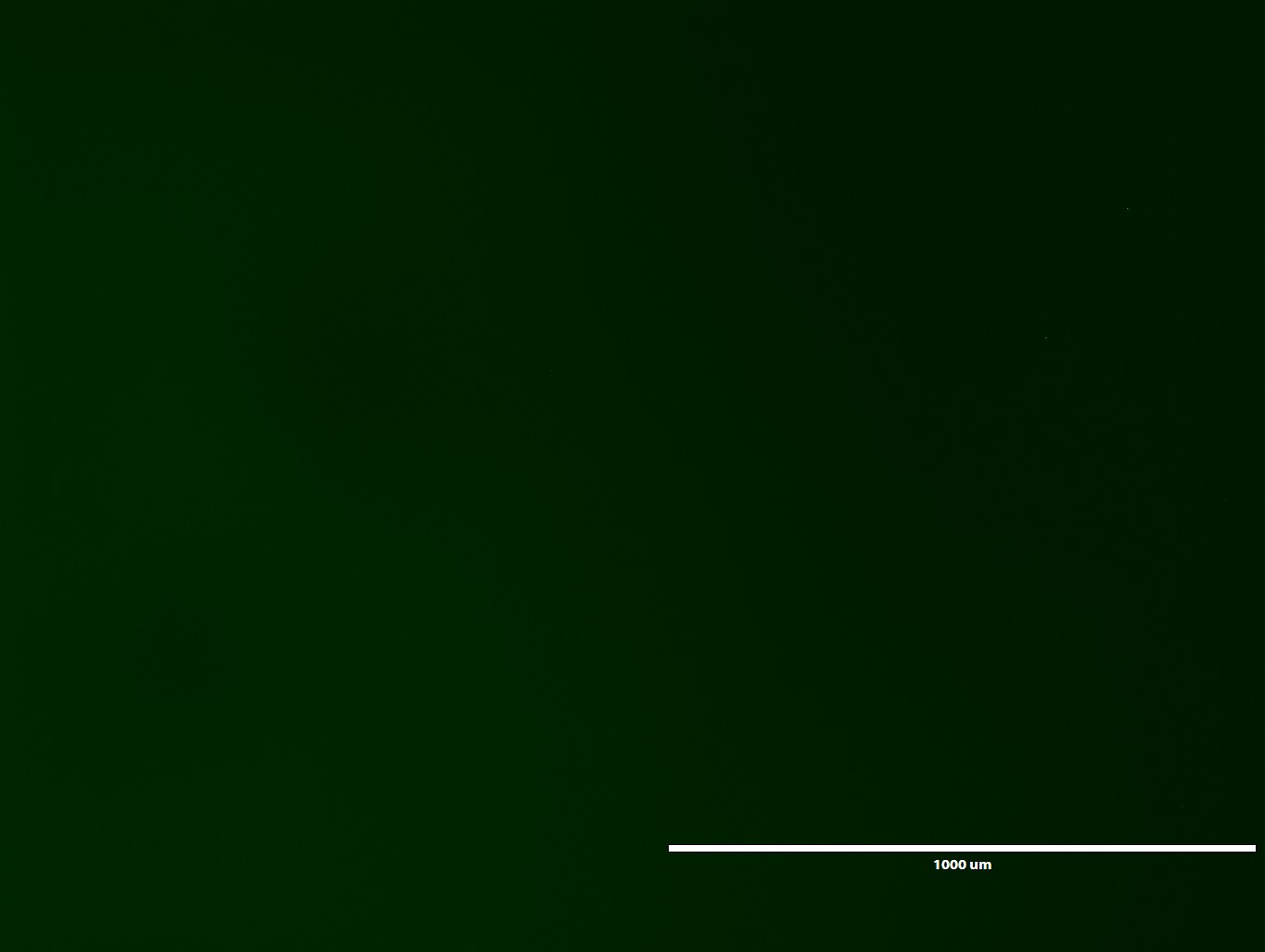

Supplement: Supplementary file 2 [file DataSheet2.ZIP › 4A raw images/NC.jpg]

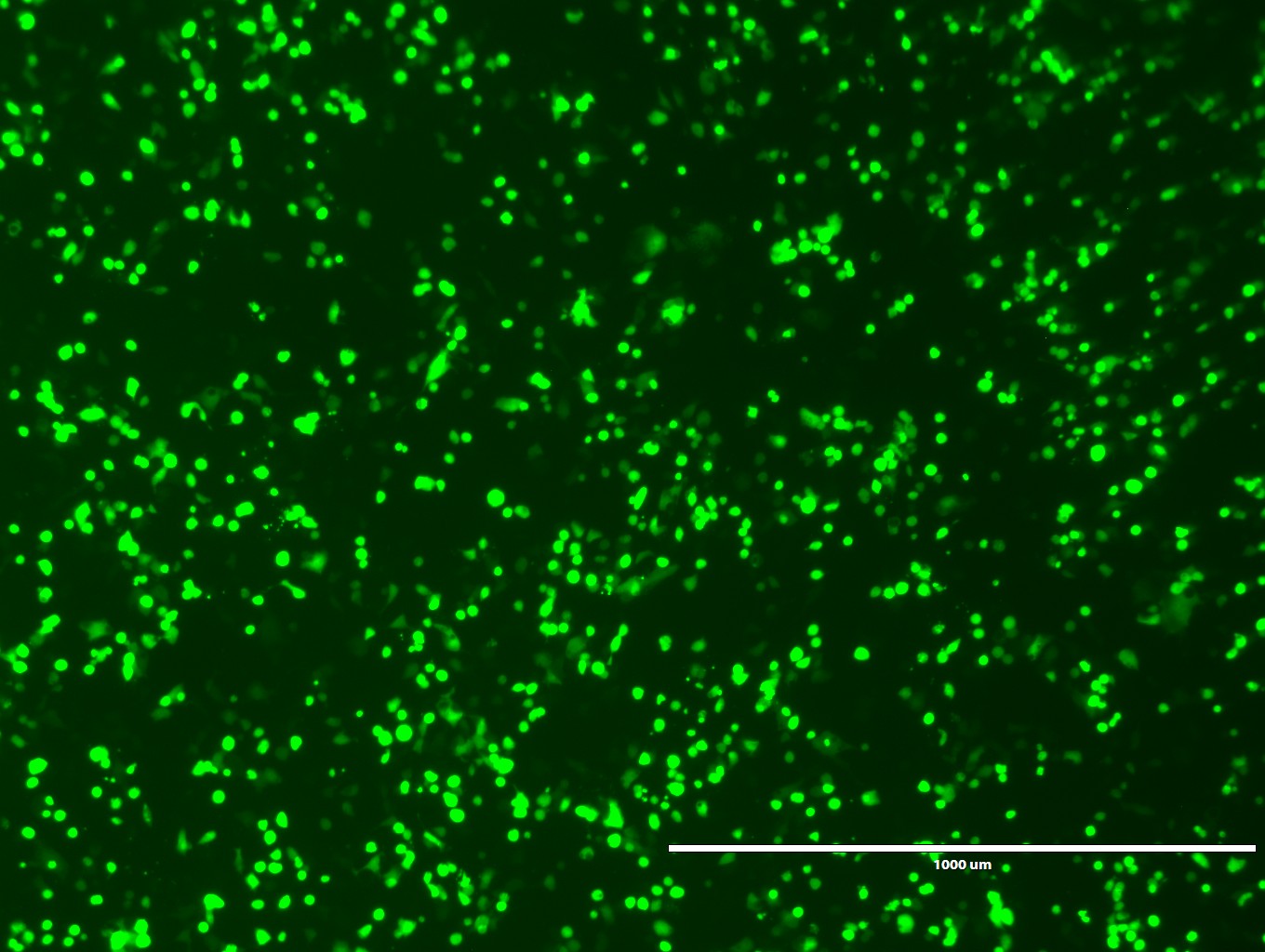

Supplement: Supplementary file 2 [file DataSheet2.ZIP › 4A raw images/WT.jpg]

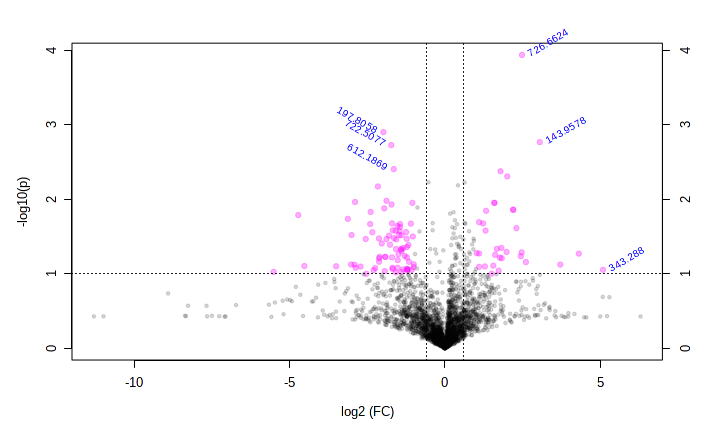

Supplement: Supplementary file 4 [file Image1.PNG]
